# Polish Adaptation of the Modified Tampa Scale of Kinesiophobia for Fatigue (TSK-F) and the Revision of the Tampa Scale in Terms of Pain for Cancer Patients

**DOI:** 10.3390/ijerph191912730

**Published:** 2022-10-05

**Authors:** Mateusz Rozmiarek, Mateusz Grajek, Ewa Malchrowicz-Mośko, Karolina Sobczyk, Karolina Krupa-Kotara, Piotr Nowaczyk, Janusz Wasiewicz, Tomasz Urbaniak, Wojciech Siejak, Urszula Czerniak, Anna Demuth, Aitor Martínez Aguirre-Betolaza, Arkaitz Castañeda-Babarro

**Affiliations:** 1 Department of Sports Tourism, Faculty of Physical Culture Sciences, Poznan University of Physical Education, 61-871 Poznan, Poland; 2 Department of Public Health, Faculty of Health Sciences in Bytom, Medical University of Silesia in Katowice, 41-902 Bytom, Poland; 3 Department of Health Economics and Health Management, Faculty of Health Sciences in Bytom, Medical University of Silesia in Katowice, 41-902 Bytom, Poland; 4 Department of Epidemiology, Faculty of Health Sciences in Bytom, Medical University of Silesia in Katowice, 41-902 Bytom, Poland; 5 Breast Cancer Unit, Breast Surgical Oncology Department, Greater Poland Cancer Center, 61-866 Poznan, Poland; 6 Department of Anthropology and Biometrics, Faculty of Physical Culture Sciences, Poznan University of Physical Education, 61-871 Poznan, Poland; 7 Department of Physical Activity and Sports, Faculty of Education and Sport, University of Deusto, 48007 Bilbao, Spain

**Keywords:** female oncology, kinesiophobia, fear of movement, breast cancer, physical activity, oncology treatment, pain, fatigue, active lifestyle, Tampa Scale

## Abstract

The aim of this study was to create a Polish adaptation of the Tampa Scale of Kinesiophobia considering fatigue, and to verify the usefulness of the scale in the context of pain in cancer patients. The study was conducted at the Breast Cancer Unit, operating at the Greater Poland Cancer Centre, and at the Poznan Centre for Specialist Medical Services in Poznan. After considering the exclusion criteria, 100 people qualified for the interviews for the final study: 50 breast cancer patients and 50 healthy respondents (without cancer). Statistical analysis of the CFA score showed that the chi-square test was not significant (χ^2^ = 10.243, *p* = 0.332), indicating an acceptable fit of items across scales. The reliability of the internal consistency of the scales was tested by examining the Cronbach’s alpha scores for each question/statement. The mean values for this indicator were 0.74 for the pain-related scale and 0.84 for the fatigue-related scale. Construct validity was confirmed for the scales; AVE for the pain-related scale was 0.64 and for the fatigue-related scale was 0.68. The results suggest the validity of examining kinesiophobia in the context of pain- and fatigue-related mobility anxiety among breast cancer patients in Poland, and that the Tampa Scale of Kinesiophobia can be adapted for different dimensions of the condition. Both versions of the scale demonstrated adequately prepared parametric constructs, and all correlations showed a statistically significant relationship (*p* < 0.05). The use of the Tampa Scale of Kinesiophobia in oncology patient studies in Poland may ultimately improve rehabilitation programs and enable the development of strategies to assist patients in supporting treatment to reduce movement anxiety.

## 1. Introduction

Fear of movement, as contrasted with ambulophobia [1], is known in the scientific literature under the term kinesiophobia and refers to an excessive, irrational and debilitating fear of performing physical movement, resulting from a sense of vulnerability to painful injury or re-injury [2]. Kinesiophobia alters the way people move and causes an adaptation of motor behavior to avoid or control pain, providing a substrate for motor passivity [3]. As demonstrated by Trost and colleagues [4], the greater the degree of kinesiophobia, the greater the level of pain, and the prevalence of kinesiophobia in persistent pain can be as high as 70% [5]. Originally, the term was applied exclusively to people suffering from back pain, but its versatility has led to use in the study of various other disorders and diseases as well; for example, for myocardial infarction [6], fibromyalgia [7], multiple sclerosis [8], chronic obstructive pulmonary disease [9], knee arthroplasty [10] or Parkinson’s disease [11]. Although there is generally no specific tool directly dedicated to assessing fear of movement, kinesiophobia is usually assessed using the Tampa Scale of Kinesiophobia [12]. The scale is also used to measure fear of movement in cancer survivors [13].

It is estimated that nearly 20 million new cancer cases and nearly 10 million cancer deaths occurred worldwide in 2020 [14]. The burden of cancer incidence and mortality is increasing rapidly year on year, due in part to population growth and an ageing population, but most importantly to the intensification of major cancer factors related to socio-economic, environmental and even lifestyle factors [15,16]. Importantly, in most cases, the diagnosis of cancer at an early stage allows for its targeted complete cure [17].

Among the extensive catalog of cancers, the most commonly diagnosed cancer according to the publication of Ferlay et al. [18], and after considering data from the World Health Organization, is breast cancer, which accounts for 12% among all cancers. In contrast, the most recent report on malignant neoplasms in Poland indicates that these diseases are the second cause of death in the country, just after cardiovascular diseases [19]. The treatment of breast cancer is complex and involves a combination of different modalities, including surgery, radiotherapy, chemotherapy, hormonal therapy or biological therapies, administered in different sequences [20]. However, it is necessary to prevent the disease by implementing different types of recommendations regarding diet, weight control, nutrition or exercise [21]. A special role is attributed to physical activity as the basis of various types of prevention and rehabilitation programmes, which makes it necessary to consider the problem of kinesiophobia not only in terms of theoretical knowledge, but above all in terms of practical knowledge and relating to specific patients or groups of patients [22]. Indeed, there is strong evidence that physical activity before, during and after diagnosis improves breast cancer outcomes, and furthermore protects against recurrence in breast cancer survivors [23]. However, the level of kinesiophobia among oncology patients dealing with breast cancer is unknown, and an adaptation and validation of the Tampa Scale of Kinesiophobia is needed to measure it.

To date, validation of the scale for cancer patients in the context of fatigue has been performed [13], but due to the fact that oncology patients may also be afraid of pain, for example in their limbs after breast surgery, it was necessary to validate the use of the original Tampa Scale of Kinesiophobia developed by Miller et al. [24] among oncology patients. The aim of this study was to create a Polish adaptation of the Tampa Scale of Kinesiophobia taking into account fatigue, and to verify the usefulness of the scale in the context of pain in breast cancer patients.

## 2. Materials and Methods

### 2.1. Study Area and Process

The study was conducted from September 2021 to December 2021 at the Breast Cancer Unit, operating at the Greater Poland Cancer Center (pol. Wielkopolskie Centrum Onkologii) in Poznan, (interviews with women with breast cancer) and at the Poznan Centre for Specialist Medical Services (pol. Poznański Ośrodek Specjalistycznych Usług Medycznych) in Poznan (interviews with women without breast cancer, who constituted the control group). A total of 120 people took part in the interviews, with the eligibility criteria including the need to answer each question asked. With this in mind, 100 subjects were selected for the final study: 50 patients with breast cancer and 50 patients in the control group, who additionally had to declare that they had no history of malignant tumors, spinal cord injury, myocardial infarction or stroke.

The study was reviewed and approved by the Bioethics Committee at the Poznan University of Medical Sciences (16 June 2021). A decision was made that the study does not have the characteristics of a medical experiment and can be conducted among cancer patients in the Breast Cancer Unit (as well as in a control group of healthy women). Written consent to conduct the study was also given by the directors of the Greater Poland Cancer Center and the Poznan Centre for Specialist Medical Services in Poznan. The interviewers, who were a group of adult women, were informed about the objectives of the study. Entering the questions was treated as an informed consent to participate in the study, although female respondents were free to withdraw from the study at any time without giving a reason or to skip answering the questions. The study was conducted in accordance with generally accepted ethical principles for the conduct of research stemming from the 1975 Declaration of Helsinki, and participants were treated ethically in accordance with the American Psychological Association’s code of ethics.

### 2.2. Research Instrument

Fear is one of the factors that represent a barrier to exercise in people with various diseases [6,8,25,26,27], but so far its impact in women with breast cancer has only been investigated by Sander et al. [28] and Can et al. [29]. A self-administered and anonymous survey questionnaire was used as the research tool, which included questions such as gender, age, education, current work situation, marital status and place of residence, as well as the standardized Tampa Scale for Kinesiophobia (TSK) tool. This is a tool consisting of 17 statements (items), and is used with patients to measure fear of moving due to the possibility of experiencing pain. Each item was assigned a 4-point Likert scale (1 = “I do not agree completely”, 2 = “I do not agree in part”, 3 = “I agree partially”, 4 = “I agree completely”). There was a minimum score of 17 and a maximum score of 68, and the higher the score, the greater the severity of kinesiophobia. The tool has also received numerous adaptations and validations, including Swedish [30], Spanish [31], Portuguese [32] and Japanese [33]. In our study, the scale in addition to its standard version for pain was adapted for use with fatigue sensations, and the internal consistency of the scales is described below.

### 2.3. Scale Translation

As part of the preparatory work, the original Tampa Scale of Kinesiophobia was translated into Polish by three independent certified translators specializing in scientific translations within the health and physical culture sciences. The translations were then analyzed in detail from a technical and factual point of view. Not only was the quality of the completed translation assessed, but also its usability in terms of language. To this end, comparisons were made between the translations; the variants that best conveyed the sense of the item proposed by the scale creators were selected. The completed translation was submitted for evaluation to 10 independent judges—specialists in health and physical culture sciences—who reviewed the adequacy of the translation into Polish and were given the chance to offer their suggestions for changes. The final Polish translation was produced, together with a summary of the original pain and fatigue scales (scale attached in the article Supplementary File S1).

### 2.4. Statistical Analysis

The collected data were coded and analyzed in STATISTICA 13.0 (Stat Soft Poland). Confirmatory factor analysis (CFA) was used in the mathematical treatment of the data, which tested the acceptability of items on both scales. Internal consistency reliability was estimated for both scales using Cronbach’s alpha coefficient. The average variance test (AVE) was used to assess the construct validity of both scales.

## 3. Results

A total of 120 people took part in the survey. Eligibility criteria for the study included the need to answer every question asked during the interview. Taking this into account, 100 people were qualified for the final study: 50 breast cancer patients and 50 healthy respondents.

Of the 50 female persons living with cancer (PLWC), the youngest was 23 years old and the oldest was 80 years old (mean u age was 53.08 ± 14.35 years). The mean body height of the PLWC was 1.62 ± 0.02 m, and the mean body weight was 71.14 ± 12.78 kg. Of the PLWC, 4% (2) had primary education, 46% (23) indicated the option of having a secondary education, and 50% (25) reported having a tertiary education. A total of 30% (15) resided in a rural area, 22% (11) a small town, 22% (11) a medium town, 26% (13) a large town. A total of 70% (35) of respondents indicated being in a relationship or married, 22% (11) were widowed or divorced and 8% (4) were single. A total of 54% (27) of respondents reported being economically active, 32% (16) were retired, 6% (3) were on a pension, 2% (1) were studying and 6% (3) were unemployed.

Of the 50 healthy women, the youngest was 23 years old and the oldest was 80 years old (mean u age was 48.78 ± 16.47 years). The mean body height of the healthy women was 1.69 ± 0.08 m, and the mean body weight was 71.40 ± 12.17 kg. Of the healthy women, 8% (4) had primary education, 34% (17) indicated the option of having a secondary education and 58% (29) reported having a tertiary education. A total of 22% (11) resided in a rural area, 2% (1) a small city, 22% (11) a medium city, 54% (27) a large city. A total of 46% (23) of respondents indicated being in a relationship or married, 24% (12) were widowed or divorced and 30% (15) were single. A total of 56% (28) of respondents reported being economically active, 22% (11) were retired, 12% (6) were on a pension, 6% (3) were studying and 4% (2) were unemployed.

The socio-demographic characteristics are presented in Table 1.

Statistical analysis of the CFA score showed that the chi-square test was not significant (χ^2^ = 10.243, *p* = 0.332), indicating an acceptable fit of items across scales. The reliability of the internal consistency of the scales was tested by examining the Cronbach’s alpha scores for each question/statement. The mean values for this indicator were 0.74 for the pain-related scale and 0.84 for the fatigue-related scale. The reliability of each factor met the recommended criterion of α = 0.70. Construct validity was confirmed for the scales; AVE for the pain-related scale was 0.64 and for the fatigue-related scale was 0.68. These exceeded the cut-off point set at 0.50. Reliability, AVE, factor loadings and *t*-values for the pain- and fatigue-related scales are presented in Table 2.

All correlations showed a statistically significant relationship (*p* < 0.05). The highest strength of correlation was shown between items: “Just because something increases my pain, it does not mean it is dangerous” vs. “If I tried to overcome this, my fatigue would increase” (H = 19.213; r = 0.881; *p* = 0.001) and “Although my condition is painful, I would be better off if I were physically active” vs. “My fatigue would probably be relieved if I were to exercise” (H = 17.458; r = 0.828; *p* = 0.001). This result indicates that the scales were satisfactorily distinct from each other, and represented valid psychometric constructs. This is shown in Table 3.

## 4. Discussion

Breast cancer is one of the most serious diseases affecting women worldwide. Research has repeatedly shown that physical activity is an important element in the prevention of breast cancer and reduces the risk of recurrence after treatment [34]. The results obtained above suggest the validity of examining kinesiophobia in the context of pain- and fatigue-related movement anxiety among breast cancer patients, and the Tampa Scale of Kinesiophobia can be adapted for different dimensions of the condition. Both versions of the scale demonstrated adequately prepared parametric constructs, and all correlations showed a statistically significant relationship (*p* < 0.05).

Physical activity among patients with breast cancer should be considered comprehensively, and its absence may prognosticate a negative impact on the patient’s health and promote a deterioration of quality of life through an increase in movement limitations and even lead to disability [35]. Indeed, patients should be made aware of the need to undertake daily physical activity, whether in the form of an adequate amount of walking, or in the form of rehabilitation, active leisure activities or sports. This is all the more so because, as Irwin et al. [36] point out, breast cancer patients decrease their physical activity in the period from diagnosis to diagnosis.

The lack of any physical activity is one of the modifiable risk factors for breast cancer, so it seems extremely important to activate patients in this area. Every effort should therefore be made to ensure that patients receive sufficient help and support in this area from the health care system. It may be worth considering the implementation of telemedicine tools in Poland, which make it possible, for example, to provide doctor or physiotherapist supervision of the physical activity process via a smartphone application, as Rabin and Bock [37] proposed.

Although breast cancer can also affect men [38], only women were involved in our study, as it is women who are diagnosed with 99% of breast malignancies. Interestingly, a negligible amount of research on kinesiophobia in breast cancer patients has been performed to date, and the only one—indicating a kinesiophobia rate of 30.8%—was performed in Turkey [29]. However, it should be noted that gender has a significant impact on kinesiophobia levels, as shown in a study by Bränström and Fahlström [39], verifying myofascial pain and seeing higher kinesiophobia scores in men than in women. This issue indicates that socio-demographic factors may be an important reference for further similar studies, although biological, psychological, social and even cultural factors also play a role [40,41]. Nevertheless, a previous study conducted by Sander et al. [28] on cancer patients indicated that a group of women engaged in regular physical activity prior to breast cancer diagnosis showed no fear of exercise. With the present study, it will be possible to verify this on other patient groups.

In addition, it is worth noting that important variables that should be the subject of further research are the issues of analgesic use for painful conditions, and its impact on perceived fear of movement. These issues, in terms of neck and back pain, were raised by researchers including Asiri et al. [42]. This aspect additionally relates to fear of falling [1] and cancer stage [43,44]. Caraceni and Shkodra [43] indicated that at least half of PLWC experience pain of hard-to-define but severe intensity in various locations of the body, and that its nature excluded them from daily functioning.

## 5. Strengths and Limitations

The results presented in the paper offer new considerations for the field of knowledge concerning kinesiophobia research. It is still a rarely studied phenomenon in Poland, and the use of scales with psychometric values permits further development in this branch of knowledge. Further studies should focus on the correlation of the obtained results with a detailed health history among PLWC; issues such as the stage of cancer or the use of agents with analgesic potential should be considered. In the current study, the size and selection of the study sample is an important asset. It is worth noting that data collection in the oncology population is sometimes problematic due to the different stages of the disease and associated psychophysical afflictions. Nevertheless, as indicated above, the authors note some shortcomings to this study, which positively set new directions for research. Adaptation of the scale to Polish conditions, with extension to fatigue issues, would certainly allow for further research.

## 6. Conclusions

The results indicate that the Tampa Scale of Kinesiophobia can be adapted for different dimensions of health status related to (breast) cancer patients. In a self-report study, its psychometric properties were demonstrated in its application to fatigue associated with anxiety about moving. Coping with kinesiophobia during and after cancer is a process that requires not only a great deal of self-denial, but also often external support. The use of the Tampa Scale of Kinesiophobia in patient studies may ultimately improve rehabilitation programmes and enable the development of strategies to assist patients in supporting treatment to reduce movement anxiety.

## Figures and Tables

**Table 1:** Socio-demographic variables of participants.

Demographic Variables	Women with Breast Cancer (*n* = 50)	Women without Breast Cancer (*n* = 50)
	(Mean ± SD)/*n* (%)	(Mean ± SD)/*n* (%)
**Age**	53.08 ± 14.35	48.78 ± 16.47
Body height	1.62 ± 0.02	1.69 ± 0.08
Body weight	71.14 ± 12.78	71.40 ± 12.17
**Education Level**		
Primary	2 (4%)	4 (8%)
Secondary	23 (46%)	17 (34%)
Higher	25 (50%)	29 (58%)
**Marital Status**		
Single	4 (8%)	15 (30%)
In a relationship or married	35 (70%)	23 (46%)
Divorced or widowed	11 (22%)	12 (24%)
Employment Status		
Student	1 (2%)	3 (6%)
Proffesionally active	27 (54%)	11 (22%)
Unemployed	3 (6%)	2 (4%)
Annuitant	3 (6%)	6 (12%)
Pensioner	16 (32%)	11 (22%)
**Place of Residence**		
City with over 100,000 inhabitants	13 (26%)	27 (54%)
City with 20,000–100,000 inhabitants	11 (22%)	11 (22%)
City with up to 20,000 inhabitants	11 (22%)	1 (2%)
Village	15 (30%)	11 (22%)

**Table 2:** Reliability, AVEs, factor loadings, *t*-values for pain and fatigue scales.

No.	Scale/Item	Alpha-Cronbach	AVE	Factor Loadings	*t*-Test
	**TSK for PAIN**				
1.	I’m afraid that I might injure myself if I exercise.	**0.74**	**0.64**	0.75	12.56
2.	If I were to try to overcome it, my pain would increase.			0.77	12.62
3.	My body is telling me I have something dangerously wrong.			0.73	12.12
4.	My pain would probably be relieved if I were to exercise.			0.67	10.58
5.	People aren’t taking my medical condition seriously enough.			0.69	10.54
6.	My accident has put my body at risk for the rest of my life.			0.66	10.21
7.	Pain always means I have injured my body.			0.81	14.32
8.	Just because something aggravates my pain does not mean it is dangerous.			0.83	14.36
9.	I am afraid that I might injure myself accidentally.			0.77	12.72
10.	Simply being careful that I do not make any unnecessary movements is the safest thing I can do to prevent my pain from worsening.			0.79	12.68
11.	I wouldn’t have this much pain if there weren’t something potentially dangerous going on in my body.			0.75	12.44
12.	Although my condition is painful, I would be better off if I were physically active.			0.71	13.81
13.	Pain lets me know when to stop exercising so that I don’t injure myself.			0.77	12.42
14.	It’s really not safe for a person with a condition like mine to be physically active.			0.69	10.88
15.	I can’t do all the things normal people do because it’s too easy for me to get injured.			0.71	12.62
16.	Even though something is causing me a lot of pain, I don’t think it’s actually dangerous.			0.81	14.26
17.	No one should have to exercise when he/she is in pain.			0.79	12.28
	**TSK for FATIGUE**				
1.	I am afraid that I might be more fatigued if I exercise.	**0.84**	**0.68**	0.83	14.68
2.	If I tried to overcome this, my fatigue would increase.			0.85	14.16
3.	My body is telling me I have something dangerously wrong.			0.77	12.18
4.	My fatigue would probably be relieved if I were to exercise.			0.77	12.22
5.	People aren’t taking my medical condition seriously enough.			0.75	12.08
6.	My accident has put my body at risk for the rest of my life.			0.67	10.58
7.	Fatigue always means I have harmed my body.			0.67	10.58
8.	Just because something increases my fatigue, it does not mean it is dangerous.			0.69	10.98
9.	I am afraid that I might make my fatigue symptoms worse accidentally.			0.65	10.86
10.	Simply being careful that I do not make any unnecessary movements is the safest thing I can do to prevent my fatigue from worsening.			0.91	16.22
11.	I wouldn’t have this much fatigue if there weren’t something potentially dangerous going on in my body.			0.75	12.46
12.	Although I am fatigued, I would be better off if I were physically active.			0.83	14.36
13.	Fatigue lets me know when to stop exercising so that I don’t harm myself.			0.75	12.12
14.	It’s really not safe for a person with a condition like mine to be physically active.			0.67	10.52
15.	I can’t do all the things normal people do because it’s too easy for me to get tired.			0.71	12.44
16.	Even though something makes me fatigued, I don’t think it’s actually harming me.			0.69	10.56
17.	No one should have to exercise when he/she is fatigued.			0.65	10.42

**Table 3:** Correlations between pain and fatigue TSK scales.

Item	1	2	3	4	5	6	7	8	9	10	11	12	13	14	15	16	17
1	1.00	-	-	-	-	-	-	-	-	-	-	-	-	-	-	-	-
2	0.51	1.00	-	-	-	-	-	-	-	-	-	-	-	-	-	-	-
3	0.34	0.43	1.00	-	-	-	-	-	-	-	-	-	-	-	-	-	-
4	0.25	0.41	0.48	1.00	-	-	-	-	-	-	-	-	-	-	-	-	-
5	0.68	0.28	0.61	0.62	1.00	-	-	-	-	-	-	-	-	-	-	-	-
6	0.62	0.59	0.55	0.63	0.63	1.00	-	-	-	-	-	-	-	-	-	-	-
7	0.66	0.54	0.48	0.52	0.58	0.53	1.00	-	-	-	-	-	-	-	-	-	-
8	0.36	0.88	0.33	0.41	0.43	0.41	0.47	1.00	-	-	-	-	-	-	-	-	-
9	0.33	0.41	0.65	0.28	0.44	0.28	0.31	0.53	1.00	-	-	-	-	-	-	-	-
10	0.65	0.28	0.45	0.59	0.61	0.59	0.51	0.33	0.51	1.00	-	-	-	-	-	-	-
11	0.45	0.59	0.43	0.54	0.55	0.54	0.45	0.65	0.34	0.41	1.00	-	-	-	-	-	-
12	0.44	0.54	0.41	0.82	0.48	0.51	0.64	0.45	0.25	0.28	0.43	1.00	-	-	-	-	-
13	0.41	0.62	0.28	0.43	0.44	0.34	0.41	0.71	0.68	0.59	0.44	0.43	1.00	-	-	-	-
14	0.70	0.63	0.59	0.41	0.33	0.25	0.53	0.68	0.62	0.54	0.61	0.41	0.36	1.00	-	-	-
15	0.61	0.47	0.54	0.28	0.65	0.68	0.61	0.45	0.66	0.28	0.55	0.28	0.33	0.53	1.00	-	-
16	0.53	0.52	0.44	0.59	0.45	0.62	0.52	0.41	0.56	0.53	0.48	0.59	0.65	0.49	0.63	1.00	-
17	0.42	0.41	0.41	0.54	0.67	0.66	0.42	0.38	0.53	0.54	0.44	0.54	0.45	0.51	0.64	0.44	1.00

## Data Availability

The raw data supporting the conclusions of this article will be made available by the authors, without undue reservation.

## Supplementary Materials

The following supporting information can be downloaded at: https://www.mdpi.com/article/10.3390/ijerph191912730/s1, the Polish translation of the Tampa Scale of Kinesiophobia for Pain and Fatigue is attached in the Supplementary File S1.
