# Supplementary material for: Polish Adaptation of the Modified Tampa Scale of Kinesiophobia for Fatigue (TSK-F) and the Revision of the Tampa Scale in Terms of Pain for Cancer Patients
Source: Int J Environ Res Public Health. 2022 Oct 5;19(19):12730. doi: 10.3390/ijerph191912730 (PMC9566366; doi:10.3390/ijerph191912730)
Supplement: Supplementary file 1 [file ijerph-19-12730-s001.zip › ijerph-1935222-supplementary.pdf]

Supplementary File S1: Polish translation of the scale based on the original TSK and TSK-F.

Item No.	The Tampa Scale – Pain (by Miller et al., 1991).	The Tampa Scale – Fatigue (by Velthuis et al., 2011).	Polish Adaptation of the Miller et al. and Velthuis et al. Scale Versions
1.		I am afraid that I might be more fatigued if I exercise.	Boję się, że będę bardziej zmęczony/a jeśli będę aktywny/a fizycznie.
	I'm afraid that I might injure myself if I exercise.		Boje się, że będę odczuwał(a) silniejszy ból jeśli będę aktywny/a fizycznie.
2.		If I tried to overcome this, my fatigue would increase.	Jeśli będę aktywny/a fizycznie, to będę bardziej zmęczony/a.
	If I were to try to overcome it, my pain would increase.		Jeśli będę aktywny/a fizycznie, to będę odczuwał(a)silniejszy ból.
3.	My body is telling me I have something dangerously wrong.	My body is telling me I have something dangerously wrong.	Mój organizm mówi mi, że coś jest z nim nie tak.
4.		My fatigue would probably be relieved if I were to exercise.	Jeśli będę aktywny/a fizycznie to będę mniej zmęczony/a.
	My pain would probably be relieved if I were to exercise.		Jeśli będę aktywny/a fizycznie to będę odczuwał(a) mniejszy ból.
5.	People aren't taking my medical condition seriously enough.	People aren't taking my medical condition seriously enough.	Ludzie nie traktują mojego stanu zdrowia wystarczająco poważnie.
6.	My accident has put my body at risk for the rest of my life.	My accident has put my body at risk for the rest of my life.	Moja choroba zagraża mojemu organizmowi do końca życia.
7.		Fatigue always means I have harmed my body.	Zmęczenie oznacza, że szkodzę swojemu ciału.
	Pain always means I have injured my body.		Ból oznacza, że szkodzę swojemu ciału.
8.		Just because something increases my fatigue, it does not mean it is dangerous.	To, że coś nasila moje zmęczenie, nie oznacza, że jest dla mnie niebezpieczne.
	Just because something aggravates my pain does not mean it is dangerous.		To, że coś nasila moje dolegliwości bólowe nie oznacza, że jest niebezpieczne.
9.		I am afraid that I might make my fatigue symptoms worse accidentally.	Boję się, że przypadkowo mogę nasilić moje objawy zmęczenia.

	I am afraid that I might injure myself accidentally.		Boję się, że przypadkowo mogę nasilić moje objawy bólowe.
10.		Simply being careful that I do not make any unnecessary movements is the safest thing I can do to prevent my fatigue from worsening.	Uważam, że niewykonywanie niepotrzebnych ruchów ciała, jest najbezpieczniejszym co mogę zrobić, aby zapobiec pogłębianiu się zmęczenia.
	Simply being careful that I do not make any unnecessary movements is the safest thing I can do to prevent my pain from worsening.		Uważam, że niewykonywanie niepotrzebnych ruchów ciała, jest najbezpieczniejszym co mogę zrobić, aby zapobiec pogłębianiu się dolegliwości bólowych.
11.		I wouldn't have this much fatigue if there weren't something potentially dangerous going on in my body.	Nie czuł(a)bym zmęczenia, gdyby nie działo się coś potencjalnie niebezpiecznego z moim ciałem.
	I wouldn't have this much pain if there weren't something potentially dangerous going on in my body.		Nie czuł(a)bym bólu, gdyby nie działo się coś potencjalnie niebezpiecznego z moim ciałem.
12.		Although I am fatigued, I would be better off if I were physically active.	Chociaż czuję się zmęczony/a byłoby lepiej, abym był(a) aktywna fizycznie.
	Although my condition is painful, I would be better off if I were physically active.		Chociaż odczuwam ból byłoby lepiej, abym był(a) aktywny/a fizycznie.
13.		Fatigue lets me know when to stop exercising so that I don't harm myself.	Zmęczenie daje mi znać, kiedy przestać ćwiczyć, aby nie zaszkodzić organizmowi.
	Pain lets me know when to stop exercising so that I don't injure myself.		Ból daje mi znać, kiedy przestać ćwiczyć, aby nie zaszkodzić organizmowi.
14.	It's really not safe for a person with a condition like mine to be physically active.	It's really not safe for a person with a condition like mine to be physically active.	Aktywność fizyczna naprawdę nie jest bezpieczna dla osoby w takim stanie zdrowia jak mój
15.		I can't do all the things normal people do	Nie mogę robić wszystkich rzeczy, które robią inni

		because it’s too easy for me to get tired.	ludzie, ponieważ zbyt łatwo się męczę.
	I can't do all the things normal people do because it's too easy for me to get injured.		Nie mogę robić wszystkich rzeczy, które robią inni ludzie, ponieważ zbyt szybko odczuwam ból.
16.		Even though something makes me fatigued, I don't think it's actually harming me.	Chociaż coś mnie męczy, nie wydaje mi się, żeby mi to szkodziło.
	Even though something is causing me a lot of pain, I don't think it's actually dangerous.		Chociaż coś sprawia mi ból, nie wydaje mi się, żeby mi to szkodziło.
17.		No one should have to exercise when he/she is fatigued.	Nikt nie powinien ćwiczyć, gdy jest zmęczony.
	No one should have to exercise when he/she is in pain.		Nikt nie powinien ćwiczyć, gdy odczuwa ból.
